# Musashi1 expression cells derived from mouse embryonic stem cells can be enriched in side population isolated by fluorescence activated cell sorter

**DOI:** 10.1186/1471-2121-12-47

**Published:** 2011-10-26

**Authors:** Tao Yu, Li-Na Zhao, Shao-Yang Lan, Miao-Jing Fan, Yu Gong, Liu Shi, Yu-Hong Yuan, Kai-Hong Huang, Qi-Kui Chen

**Affiliations:** 1 Department of Gastroenterology, the Second Affiliated Hospital, Sun Yat-Sen University, 107 Yan Jiang Xi Road, Guangzhou, Guangdong, People's Republic of China; 2 Department of Gastroenterology, the First Affiliated Hospital of Guangzhou University of Chinese Medicine, 16 Ji Chang Road, Guangzhou, Guangdong, People's Republic of China; 3 Department of Pathology, the Second Affiliated Hospital, Sun Yat-Sen University, 107 Yan Jiang Xi Road, Guangzhou, Guangdong, People's Republic of China; 4 Department of Internal Medicine, Hubei Xinhua Hospital, 5 Xin Tian Men Dun Road, Wuhan, Hubei, People's Republic of China; 5 Department of Gastroenterology, the First People's Hospital, 69 Tai Gong Road, Ganzhou, Jiangxi, People's Republic of China

## Abstract

**Background:**

Purifying stem cells is an inevitable process for further investigation and cell-therapy. Sorting side population (SP) cells is generally regarded as an effective method to enrich for progenitor cells. This study was to explore whether sorting SP could enrich for the Musashi1 (Msi1) positive cells from Msi1 high expression cells (Msi1^high ^cells) derived from mouse embryonic stem cells (ESCs) in vitro.

**Results:**

In this study, Msi1^high ^cell population derived from ESCs were stained by Hoechst 33342, and then the SP and non-SP (NSP) fractions were analyzed and sorted by fluorescence activated cell sorter. Subsequently, the expressions of Msi1 and other markers for neural and intestinal stem cells in SP and NSP were respectively detected. SP and NSP cells were hypodermically engrafted into the backs of NOD/SCID mice to form grafts. The developments of neural and intestinal epithelial cells in these grafts were investigated. SP fraction was identified and isolated from Msi1^high ^cell population. The expression of Msi1 in SP fraction was significantly higher than that in NSP fraction and unsorted Msi1^high ^cells (*P*< 0.05). Furthermore, the markers for neural cells and intestinal epithelial cells were more highly expressed in the grafts from SP fraction than those from NSP fraction (*P*< 0.05).

**Conclusions:**

SP fraction, isolated from Msi1^high ^cells, contains almost all the Msi1-positive cells and has the potential to differentiate into neural and intestinal epithelial cells in vivo. Sorting SP fraction could be a convenient and practical method to enrich for Msi1-positive cells from the differentiated cell population derived from ESCs.

## Background

Embryonic stem cells (ESCs) are pluripotent cells derived from the inner cell mass of the mammalian blastocyst with self-renewal capacity and multi-developmental plasticity, which makes ESCs a powerful tool for cell-based therapy [1,2]. Several lines of evidence confirmed that under appropriate conditions, ESCs could be induced to differentiate into pancreatic beta-cells, liver cells, myocardial cells, hematopoietic cells, and neural stem cells [3-9]. However, these strategies generally produced the desired cells only within heterogeneous cell populations, including pluripotent stem cells and undesired ESC derivatives. The transplantation of the cell population into tissues is inevitably associated with formation of teratomas, impeding the application of ESC-based therapies in clinic [10-12]. Recent studies revealed that the formation of teratomas was not observed after transplanting purified progenitor cells derived from ESCs [13-17]. These findings indicated that differentiating and purifying ESC-derived cells in vitro could be a strategy that renders ESCs safe and effective in clinic.

It has been reported that mouse ESCs had the potential to differentiate into a gut-like structure and neural cells in vitro [18,19]. Musashi1 (Msi1), expressed in the cytoplasm and nucleus of cells, is an important marker for intestinal epithelial stem cells (IESCs) and neural stem cells (NSCs) [20-22]. In our recent study, we had found that Msi1 and hairy and enhancer of split 1 (Hes1) high-expression cells (Msi1^high^Hes1^high ^cells) derived from mouse ESCs could develop into small intestinal epithelial cells, which enhanced the repair of small intestinal injury in vivo [23]. Furthermore, to purify the Msi1-positive cell, we constructed a GFP reporter plasmid vector driven by Msi1-specific promoter (pMsi1-GFP vector) [24]. Although the pMsi1-GFP vector can be used to mark Msi1-positive cells from a cell population, the isolation process is quite complicated and depends on a cell transfection technique, which restricts its application. Therefore, a more convenient and practical separation strategy should be established for the further investigation of Msi1-positive cells.

Side population (SP) analysis, a widely used flow cytometry assay, based on the ability of cells to efflux fluorescent DNA-binding dye Hoechst 33342, can identify stem cells in tissues, and is a method which opens up the potential to further enrich stem cells within heterogeneous populations [25]. SP was first identified and sorted from bone marrow by fluorescence activated cell sorter (FACS) as a distinct cell population highly enriched for hematopoietic stem cells and endowed with long-term repopulating capacity [26]. Since this discovery, an increasing number of studies have shown that an analogous SP fraction has been identified in a variety of tissues with high levels of stem-like gene expression and multipotent differentiation potential [27-35]. The use of SP analysis was also described to isolate a putative stem cell population from mouse small intestine, and Msi1 was highly expressed in the SP fraction [36]. This suggested that SP sorting could be an effective method to enrich for progenitor cells, especially in the absence of definitive cell-surface marker.

In this study, our objective was to establish a practical process to enrich for the Msi1 positive cells from Msi1 high-expression cell (Msi1^high ^cell) population derived from mouse ESCs in vitro.

## Methods

### Maintenance of mouse ESCs and embryonic bodies (EBs) formation

The mouse ESC line, ES-E14TG2a (40, XY) was maintained without feeder cells in Dulbecco's Modified Eagle Medium (DMEM; high glucose; GIBCO BRL, USA) supplemented with 10% fetal calf serum (FCS; Hyclone, USA), 10 mM HEPES (GIBCO BRL, USA), 0.12% sodium bicarbonate, 0.1 mM nonessential amino acids (Hyclone, USA), 0.1 mM 2-mercaptoethanol (2ME; GIBCO BRL, USA), 100 U/mL penicillin G, 100 μg/mL streptomycin, and 1000 U/mL leukemia inhibitory factor (LIF; Chemicon, USA). Subsequently, ESCs were cultured by the hanging-drop method (32 μl per drop) to form EBs at a concentration of 1 × 10^6 ^cells/ml in EB medium that consisted of high glucose DMEM supplemented with 10% FCS, 10 mM HEPES, 0.12% sodium bicarbonate, 0.1 mM nonessential amino acids, 0.1 mM 2ME, 100 U/mL penicillin G, and 100 μg/mL streptomycin. ESC and EB cultures were maintained in a humidified chamber in a 5% CO_2_-air mixture at 37°C.

### Differentiation of Msi1^high ^cells

Five-day EB cells were dissociated with trypsin (0.25%)/EDTA and seeded on 6-well culture plates (Nunc, USA) with a concentration of 1 × 10^5 ^cells/well. Subsequently, the cultured EB cells were induced by a serum-free medium (EGF medium) that consisted of high glucose DMEM supplemented with 10% Knockout™ serum replacement (KSR; Invitrogen Corporation, USA), 40 ng/mL EGF (CHEMICON International, USA), 10 mM HEPES, 0.12% sodium bicarbonate, 0.1 mM nonessential amino acids, 0.1 mM 2ME, 100 U/mL penicillin G, and 100 μg/mL streptomycin. The dissociated EB cells synchronously cultured in serum-free control medium without EGF (SF medium) and DMEM medium with 10% FCS (FCS medium) were treated as control groups.

### Real-time quantitative RT-PCR

Total RNA was extracted using TRIzol^® ^Reagent (Invitrogen Corporation, USA). The concentration of isolated total RNA was calculated from the absorbance at 260 nm obtained using a UV-2450 spectrophotometer (Shimadzu, Japan). To generate cDNA, 1 μg of total RNA was reverse-transcribed using a ReverTra Ace-α-^® ^kit (Toyobo Bio-Technology, Japan). Real-time PCR was performed using a Real-time™ PCR Master Mix kit (Toyobo Bio-Technology, Japan) on a Rotor-Gene 6000 detector (Corbett Research, Mortlake, Australia) according to the manufacturer's instructions. The primers were designed (forward and reverse): mouse Msi1, 5'- TAG TTC GAG GGA CAG GCT CT-3' and 5'- GTT GAG GGA CAG GCA GTA GC-3'; mouse Hes1, 5'- GGA GAG GCT GCC AAG GTT TT-3' and 5'- GCA AAT TGG CCG TCA GGA-3'; mouse leucine rich repeat containing G protein coupled receptor 5 (Lgr5), 5'- CAC CAG CTT ACC CCA TGA CT-3' and 5'- CTC CTG CTC TAA GGC ACC AC-3'; mouse achaete-scute complex homolog 2 (Ascl2), 5'- GGT GAC TCC TGG TGG ACC TA-3' and 5'- TCC GGA AGA TGG AAG ATG TC-3'; mouse Bmi1, 5'- TGT CCA GGT TCA CAA AAC CA-3' and 5'- TGC AAC TTC TCC TCG GTC TT-3'; mouse Nestin, 5'- CCA GAG CTG GAC TGG AAC TC-3' and 5'- ACC TGC CTC TTT TGG TTC CT-3'; mouse SRY-box containing gene 2 (Sox2), 5'- AAG GGT TCT TGC TGG GTT TT-3' and 5'- AGA CCA CGA AAA CGG TCT TG -3'; mouse 18S ribosomal RNA, 5'- GCT AGG AAT AAT GGA ATA GG-3' and 5'- ACT TTC GTT CTT GAG GAA TG-3'. Data were analyzed using the ΔΔCt method with 18S ribosomal RNA as the constitutive marker [37].

### Immunocytochemistry for Msi1

The immunostaining for Msi1 was performed using an UltraSensitive™ S-P kit (Maxin, Fuzhou, China). The fixed cells were treated with normal goat serum for 15 min at 37°C and then were incubated with rabbit anti-mouse Msi1 polyclonal antibody (CHEMICON International, USA) at a 1:200 dilution. Cells were incubated with biotin-conjugated secondary antibody for 20 min at 37°C, and streptavidin-alkaline phosphatase complex was applied for 15 min at 37°C after a wash with PBS. After a 15 min PBS wash, the sections were subsequently incubated in 3,3'-diaminobenzidine tetrahydrochloride (DAB; Boster, Wuhan, China) with 0.05% H_2_O_2 _for 5 minutes, and counterstained with hematoxylin for 12 seconds.

### Analysis and sorting of SP fraction in Msi1^high ^cells

SP analysis of the differentiated cells cultured in SF-EGF medium, FCS medium and SF medium was performed using the Hoechst 33342 staining method by FACS outlined by Goodell et al and Park et al [38,39]. The detected cells were resuspended in 0.1 M PBS containing 2% FCS at a density of 1 × 10^6 ^cells/mL and incubated with 5 μg/mL Hoechst 33342 (Sigma-Aldrich, USA) for 90 minutes at 37°C. To determine the verapamil-sensitive SP cells, partial cells were preincubated with verapamil (50 μM) for 5 minutes before the addition of Hoechst 33342 dye. Immediately after staining, the cells were centrifuged at 1000 rpm for 5 minutes at 4°C and resuspended in ice-cold 0.1 M PBS containing 2% FCS to a concentration of 1 × 10^6 ^cells/100 μL. After resuspending, propidium iodide (PI; Sigma-Aldrich, USA) was added at 2 μg/mL to gate out dead cells, and the cells were kept at 4°C until analysis and sorting. A 350 nm argon laser was used to excite Hoechst 33342 and PI. The cells were analyzed on a Beckman Coulter EPICS ALTRA cytosorter (Beckman Coulter, USA) at 405/30 nm (Hoechst blue) and 670/30 nm (Hoechst Red) according to the method described previously by Goodell et al [38]. Cells were then displayed in a Hoechst Blue versus Hoechst Red dot plot to visualize the SP cells. The SP fraction was identified and selected by gating on the characteristic emission fluorescence profile of SP cells. Data were recorded using EXPO32 MultiCOMP v1.1C and analyzed using EXPO32 analysis v1.2B. Sorted SP and non-side population (NSP) fractions were recovered in 0.1 M PBS with 10% FCS for subsequent investigation.

### RT-PCR analysis

Total RNA was extracted from tissues or cells using TRIzol^® ^Reagent according to the manufacturer's protocol. Total RNA (2 μg) was reverse-transcribed into first-strand cDNA with Oligo d(T)18 primers (TaKaRa Bio Inc., Tokyo, Japan) using a PrimeScript™ 1st Strand cDNA Synthesis kit (TaKaRa Bio Inc., Tokyo, Japan). PCR was performed with TaKaRa Ex Taq HS (TaKaRa Bio Inc., Tokyo, Japan) in PCR buffer and 0.5 μM dNTPs. The PCR cycling conditions were as follows: 1 cycle of 94°C for 1 minute; 35 cycles of 94°C for 30 seconds, 59°C for 30 seconds, 72°C for 1 minute; and 1 cycle of 72°C for 10 minutes. β-actin was used as the invariant control.

The sequences of primers used in this study are as follows (forward and reverse): mouse β-actin, 5'-GTC CAC CTT CCA GCA GAT GT-3' and 5'-CCT GGG CCA TTC AGA AAT TA-3'; mouse ATP-binding cassette transporter G2 (ABCG2), 5'-TCG CAG AAG GAG ATG TGT TG-3' and 5'-TTG GAT CTT TCC TTG CTG CT-3'; mouse Nestin, 5'- GAG AAG ACA GTG AGG CAG ATG AGT TA -3' and 5'- GCC TCT GTT CTC CAG CTT GCT -3'; mouse Tubulin β III, 5'- CTT CGG GCA GAT CTT CAG AC -3' and 5'- AGT CAA CCA GCT CTG CAC CT -3'; mouse sucrase-isomaltase (SI), 5'-GGG TCC AGC TTT TAT GGT GA-3' and 5'-TAT GTG TTC TGT GCC GGT TC-3'; mouse fatty acid binding protein 2 (Fabp2), 5'- CAC AGC TGA GAT CAT GGC ATT C -3' and 5'- CCA TCC TGT GTG ATT GTC AGT TTC -3'; mouse trefoil factor 3 (Tff3), 5'-CTC TGT CAC ATC GGA GCA GTG T-3' and 5'-TTG GCC ACC ATC AGC AGC AG-3'; mouse lysozyme 1 (Lyz1), 5'-GCA GTG CTC TGC TGC AGG AT-3' and 5'-GTC AGA CTC CGC AGT TCC GA-3'; mouse Chromogranin A (ChgA), 5'-CTG ACC GCT CCA TGA AGC TCT-3' and 5'-CCT ACT CGA GCA GCA GTC T-3'. The integrated intensity for the bands was determined by scanning densitometry and analyzed by Glyko BandScan 5.0. The data were analyzed using relative intensity with β-actin as the constitutive marker.

### Western blots analysis

The samples for Western blots analysis were sorted SP and NSP cells by FACS. All cells were incubated in RIPA lysis buffer: 50 mM Tris, 150 mM NaCl, 1% Triton X-100, 1% sodium deoxycholate, 0.1% SDS, 2 mM EDTA, and protease inhibitors (pH 7.4). Total protein in the supernatant of the cell lysate was measured by BCA Protein Assay Kit (Beyotime Institute of Biotechnology, Haimen, China). Protein (40 μg per sample) was separated by SDS-PAGE with a 12% polyacrylamide gel. The protein was transferred electrophoretically onto a PVDF membrane and incubated primary antibodies diluted in blocking buffer (5% milk powder, 0.1% Tween 20 in TBS) as follows: rabbit anti-mouse Msi1 antibody (1:500, CHEMICON International, USA) and rabbit anti-mouse β-actin antibody (1:1000, Cell Signaling Technology, MA, USA). Secondary antibodies were horseradish peroxidase-conjugated anti-rabbit antibody (1:2000). Msi1 and β-actin protein were detected by ECL chemiluminescence system. The integrated intensity for the protein bands was determined by scanning densitometry and analyzed by Glyko BandScan 5.0. The data were analyzed using relative intensity with β-actin as the constitutive marker.

### Grafts

The suspended SP and NSP cells sorted by FACS (1.5 × 10^6 ^per administration) were hypodermically engrafted into the backs of NOD/SCID mice (Laboratory Animal Center of Sun Yat-Sen University, Guangzhou, China). All experimental procedures involving mice in this study were approved by the Animal Ethics Committee of the second affiliated hospital of Sun Yat-Sen University. When the hypodermic grafts were generated, the mice were sacrificed and the grafts were investigated by histological, immunohistochemical, and RT-PCR analysis.

### Immunohistochemistry

The grafts were removed from NOD/SCID mice and fixed with 4% paraformaldehyde overnight at 4°C, embedded in paraffin, and cut at a thickness of 6 μm. The sections were placed in 0.01 M citrate buffer (pH 6.0) and treated in the microwave oven for 10 min to facilitate antigen retrieval. Following treatment with 3% H_2_O_2_, sections were placed in methanol for 10 min to quench endogenous peroxidase activity, and the immunostaining was performed using an UltraSensitive™ S-P kit (Maxin, Fuzhou, China). All sections were incubated with normal goat serum for 10 min at room temperature, and then were incubated overnight at 4°C with rabbit anti-mouse Tubulin β III (2.5 mg/mL in PBS; Epitomics, USA) and rabbit anti-mouse Fabp2 (5 mg/mL in PBS; Abcam, Cambridge, UK), respectively. Subsequently, the sections were incubated with biotin-conjugated secondary antibody for 12 min at room temperature. After a PBS wash, a streptavidin-peroxidase complex was applied for 10 min at room temperature. After a 15 min PBS wash, the sections were subsequently incubated in DAB (Boster, Wuhan, China) with 0.05% H_2_O_2 _for 6 minutes, and counterstained with hematoxylin for 30 seconds.

### Statistical analysis

All analyses were performed with a statistical software package (SAS 8 for Windows; SAS Institute; Cary, NC, USA). All data in this experiment were presented as the mean ± standard error (SE). Data were evaluated by one-way ANOVA in which multiple comparisons were performed by using the method of least significant difference. Differences were considered significant if the probability of the difference occurring by chance was less than 5 in 100 (*P*< 0.05).

## Results

### Differentiation of Msi1^high ^cells in vitro

ESCs were cultured by the hanging-drop method in EB medium to attain EBs in vitro. On the fifth day of EBs formation, the dissociated EB cells were adhesively cultured in EGF medium to induce the differentiation of Msi1^high ^cells.

**Figure 1:**
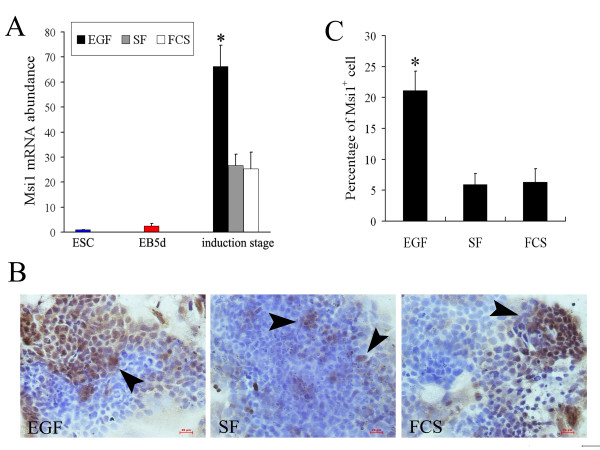
**Specification of Msi1^high ^cells in vitro derived from mouse ESCs**. (A): Relative abundance of mRNA of Msi1 in 5-day EBs (EB, red bar) and induction stage cells at 5-day in EGF medium (EGF, solid bar), SF medium (SF, gray bar), and FCS medium (FCS, blank bar) compared with ESCs (ESC, blue bar). Bars show mean ± SE (n = 3) measured by real-time RT-PCR. The red bar indicates that its abundance is 1.0 on these two scales. (*P< 0.05 compared with all other samples) (B): The immunostaining for Msi1 by immunocytochemistry was detected in partial cells at the 5-day differentiation cultured in EGF, SF, and FCS medium. (arrowheads; Bar indicates 20 μm in this panel) (C): Percentage of Msi1^+ ^cells at the 5-day differentiation cultured in EGF, SF, and FCS medium. (Bar shows mean ± SE in this panel; n = 3 individual experiments; *P< 0.05 compared with all other samples).

Real-time quantitative RT-PCR analysis suggested that Msi1 was expressed at a low level in ESCs and in 5-day EBs (Figure 1A). During the induction stage of Msi1^high ^cells, the mRNA expression of Msi1 showed an increasing trend. In the 5-day induction stage under EGF administration, Msi1 mRNA was 66.29 ± 8.38-fold greater compared with ESCs, and was significantly higher than that in the other two control groups (SF and FCS groups; Figure 1A; *P*< 0.05).

Immunocytochemistry results revealed that a portion of cells in 5-day induction stage were immunostained for Msi1 (Figure 1B). In the EGF group, the positive cells (Msi1^+ ^cells) were mainly detected in the middle of cell clones. The percentages of Msi1^+ ^cells in EGF, SF, and FCS groups were 21.1% ± 3.11%, 5.93% ± 1.75%, and 6.3% ± 2.17%, respectively (Figure 1C). The percentage of Msi1^+ ^cells in the EGF group was significantly higher than that in two control groups (Figure 1C, P < 0.05). These results indicated that 5-day administration of EGF (40 ng/mL) could induce the differentiation of Msi1^high ^cells in vitro.

### SP and NSP portions can be detected in induction stage population

**Figure 2:**
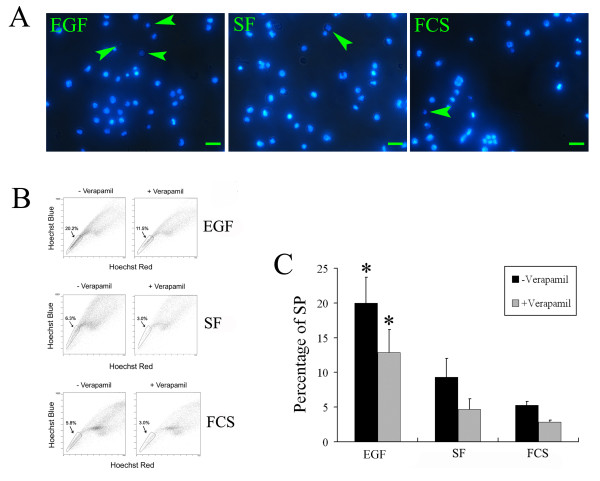
**Identification of SP and NSP fractions in Msi1^high ^cell population by FACS**. (A): A part of cells in Msi1^high ^cell population (EGF) and control groups (SF and FCS) were presented a relative weak staining after Hoechst 33342 dye. (green arrowheads; Bar indicates 50 μm in this panel) (B): FACS analysis showed a typical profile of dual-wavelength FACS of viable cells based on the Hoechst fluorescence. The distinct SP and NSP fractions were presented in all three groups (EGF, SF, and FCS). The detected cell populations in right row were treated with 50 μM verapamil (+Verapamil). (C): Percentage of SP cells in Msi1^high ^cells and two control groups (SF and FCS) with or without verapamil pretreatment (+Verapamil and -Verapamil). (Bar shows mean ± SE in this panel; n = 3 individual experiments; *P< 0.05 compared with all other groups).

To enrich for the Msi1 positive cells, the Msi1^high ^cells were stained with Hoechst 33342. Subsequently, the SP fraction was investigated by FACS. The results revealed that a portion of the cells in the Msi1^high ^cell population and control groups were stained weakly (Figure 2A). Subsequently, prepared cells were analyzed by FACS and the typical results of dual-wavelength FACS of viable cells based on the Hoechst fluorescence are shown in Figure 2B. As can be seen in Figure 2B (left row), a distinct SP and NSP were presented in cells from all three groups. The percentage of SP in the induction stage cells after 5 days of culture was 19.97% ± 3.76% for EGF (Msi1^high ^cells), 9.33% ± 2.71% for FS, and 5.3% ± 0.5% for FCS medium (Figure 2C). Based on statistical analysis, the SP percentage in Msi1^high ^cells was significantly higher than that in SF (*P *= 0.016 compared with EGF group) and FCS control groups (*P *= 0.003 compared with EGF group). Although the average percentage of SP in SF group was somewhat higher than that of FCS cell population, there was no statistically difference between them (*P *= 0.064).

Previous reports describing SP fractions from liver, mammary gland, and lung have revealed that the SP phenotype is dependent on efflux of Hoechst 33342 by multidrug resistance-like pumps, such as ABCG2/BCRP1 [32,33,40,41]. To investigate whether or not the SP from differentiated populations derived from ESCs was caused by analogous efflux of Hoechst 33342, we treated the detected cell preparations with 50 μM verapamil to inhibit members of the multi-drug resistance family. This treatment resulted in a 35.9% ± 6.8% reduction of cells sorting to SP position in EGF group (Msi1^high ^cells), and the reduction percentage in SF and FCS control groups were 49.8% ± 5.9% and 46.5% ± 2.7%, respectively (Figure 2B, right row). The percentage of SP reduced by verapamil administration in Msi1^high ^cells was significantly higher than that of other two control groups (Figure 2C, P < 0.05).

### Msi1 positive cells can be enriched in SP fraction from Msi1^high ^cell population

**Figure 3:**
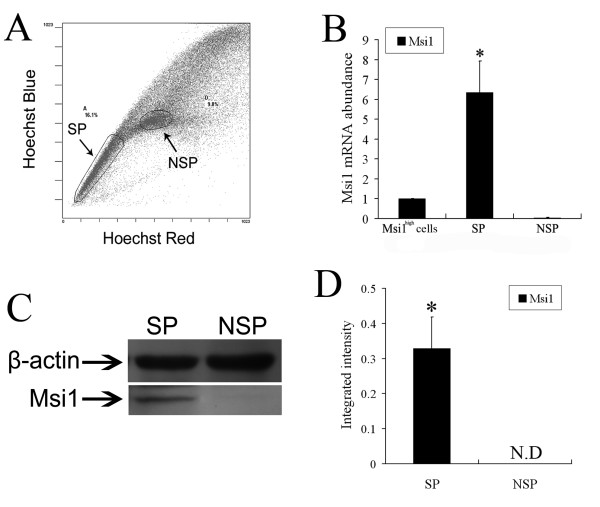
**Msi1 expression cells can be enriched in SP fraction**. (A): SP and NSP fractions were synchronically sorted from the Msi1^high ^cells by FACS. (B): Relative abundance of mRNA of Msi1 in SP and NSP fractions sorted from Msi1^high ^cells compared with unsorted Msi1^high ^cells. Bars show mean ± SE (n = 3) measured by real-time RT-PCR. The mRNA abundance in unsorted Msi1^high ^cells is 1.0 on these two scales. (*P< 0.05 compared with all other samples) (C): Expressions of the Msi1 and β-actin proteins in SP and NSP fractions sorted from Msi1^high ^cells detected by Western blots. (D): Protein levels of Msi1 indicated by the integrated intensities of corresponding bands in panel C (Mean ± SE values, n = 3 individual experiments). (*P< 0.05 compared with all other samples; N.D indicates not detected).

To explore the hypothesis that Msi1 positive cells can be enriched in SP fraction from Msi1^high ^cells, the SP and NSP cells were synchronously isolated from Msi1^high ^cell population under a condition without verapamil (Figure 3A).

Subsequently, as a gene marker for IESCs and NSCs, the expression of Msi1 was respectively detected in SP and NSP fractions on mRNA and protein levels. The results of quantitative RT-PCR revealed that the relative Msi1 mRNA abundance in SP and NSP was 6.34 ± 1.58-fold and 0.05 ± 0.02-fold greater compared with unsorted Msi1^high ^cells (Figure 3B). Western blot analysis was used to compare band intensities of Msi1 with β-actin as a constitutive marker. Msi1 protein was detected in SP and NSP fractions (Figure 3C). Integrated intensity of Msi1 protein in SP fraction was 0.329 ± 0.091, and the Msi1 expression band was not detected in NSP fraction (Figure 3D). The expression of Msi1 in SP fraction was significantly higher than that in NSP fraction and unsorted Msi1^high ^cells (*P *< 0.05). Taken together these results indicated that sorting SP fraction by FACS could be a valuable method to isolate Msi1^+ ^cells.

### Expressions of other marker genes for IESCs and NSCs

**Figure 4:**
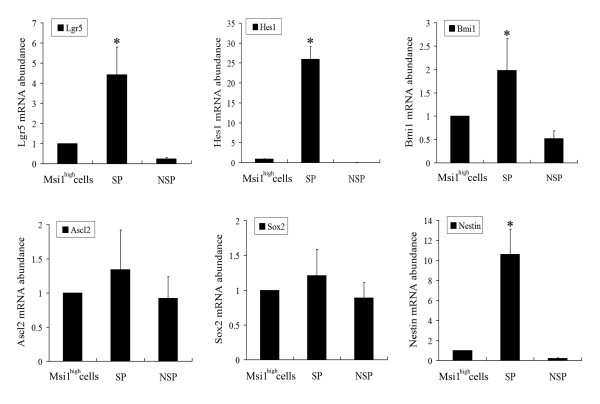
**Expressions of other markers for IESCs and NSCs in SP, NSP, and unsorted Msi1^high ^cells**. Relative abundance of mRNA of Lgr5 (A), Hes1 (B), Bmi1 (C), Ascl2 (D), Sox2 (E), and Nestin (F) in SP and NSP fractions compared with unsorted Msi1^high ^cells. Bars show mean ± SE (n = 3) measured by real-time RT-PCR. The mRNA abundance in unsorted Msi1^high ^cells is 1.0 on these scales. (*P< 0.05 compared with all other samples).

The expressions of other marker genes for IESCs (Lgr5, Hes1, Bmi1, and Ascl2) and NSCs (Sox2 and Nestin) in SP and NSP fractions were further investigated [42-46]. The results of quantitative RT-PCR revealed that the relative mRNA abundance compared with unsorted Msi1^high ^cells in SP and NSP fractions were 4.42 ± 1.38-fold and 0.25 ± 0.06-fold greater for Lgr5 (Figure 4A); 26.05 ± 3.08 folds and 0.09 ± 0.02-fold for Hes1 (Figure 4B); 1.98 ± 0.68-fold and 0.52 ± 0.17-fold greater for Bmi1 (Figure 4C); 1.34 ± 0.58-fold and 0.92 ± 0.32-fold greater for Ascl2 (Figure 4D); 1.21 ± 0.38-fold and 0.89 ± 0.22-fold greater for Sox2 (Figure 4E); and 10.62 ± 2.49-fold and 0.22 ± 0.05-fold greater for Nestin (Figure 4F). The expressions of Lgr5, Hes1, Bmi1, and Nestin in SP fraction were significantly higher than those in NSP fraction and unsorted Msi1^high ^cells (*P*< 0.05).

### ABCG2 expression in SP and NSP fractions from Msi1^high ^cells

**Figure 5:**
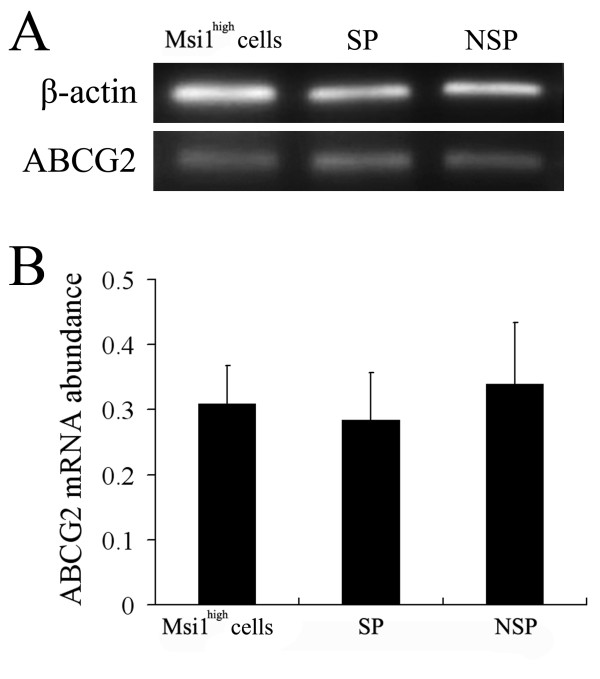
**ABCG2 expression in SP and NSP fractions from Msi1^high ^cells**. Total RNA was extracted from SP, NSP, and unsorted Msi1^high ^cells and analyzed by RT-PCR for ABCG2. (A): RT-PCR analysis for ABCG2. β-actin served as the control. (B): Relative mRNA expression of ABCG2 indicated by the integrated intensity of the corresponding bands in Panels A are shown in this panel. There was no statistical differentiation in ABCG2 expression among these three groups. (Mean ± SE; n = 3 individual experiments).

Subsequently, total RNA was extracted from SP, NSP, and unsorted Msi1^high ^cells, and analyzed by RT-PCR for ABCG2 (Figure 5A). The relative abundance of ABCG2 mRNA expression was 0.3093 ± 0.0579 in Msi1^high ^cells, 0.2844 ± 0.0726 in SP group, and 0.3397 ± 0.0938 in NSP group (Figure 5B). There was no statistical difference among these three groups in ABCG2 expression. These results indicated that a similar expression of ABCG2, a multidrug resistance-like pump, was detected in SP and NSP fractions sorted from Msi1^high ^cells.

### SP from Msi1^high ^cells can develop into intestinal epithelial and neural tissues in vivo

**Figure 6:**
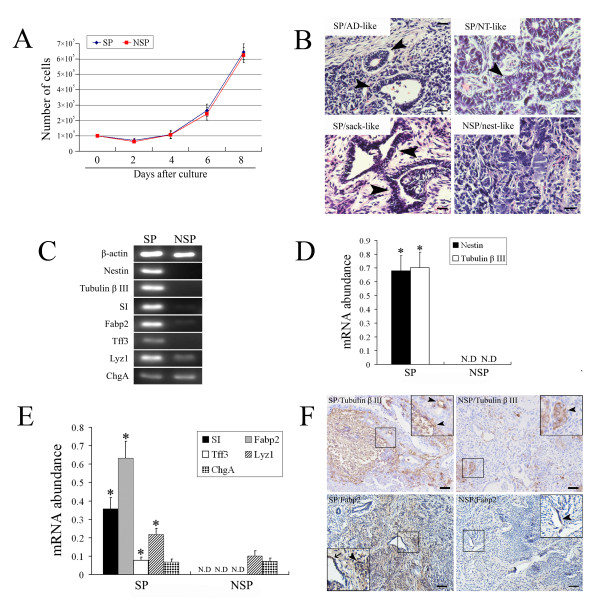
**SP cells sorted from Msi1^high ^cells could develop into neural and intestinal epithelial tissues**. (A): Proliferative curves of SP and NSP cells cultured in vitro. (B): Developmental potentials of SP and NSP cells in vivo. Abundant AD-like, NT-like, and sack-like structures were observed in the grafts from SP cells. Fibrous tissues and nest-like structures were observed in NSP grafts. Bar indicates 25 μm in this panel. (arrowheads; H&E staining) (C): RT-PCR analysis for the markers of neural cells (Nestin and Tubulin β III) and intestinal epithelium cells (SI, Fabp2, Tff3, Lyz1, and ChgA). (D): Relative mRNA expressions of Nestin and Tubulin β III are shown in this panel. (Mean ± SE; n = 3 individual experiments; *P< 0.05 compared with NSP group; N.D indicates not detected) (E): Relative mRNA expressions of SI, Fabp2, Tff3, Lyz1, and ChgA indicated by the integrated intensity in Panels C. (Mean ± SE; n = 3 individual experiments; *p < 0.05 compared with NSP group; N.D indicates not detected) (F): Immunostaining for Tubulin β III and Fabp2 in SP and NSP grafts. Tubulin β III positive cells were located in some of non-specific structures and neural tube-like structures (arrowheads). More Fabp2 positive cells were detected in SP grafts (arrowheads). Partial Fabp2 positive cells formed sack-like structures with a monolayer cell structure (arrow). The special portion was zoomed and magnified. Bar indicates 50 μm in Tubulin β III panels and 100 μm in Fabp2 panels.

The SP and NSP cells sorted from Msi1^high ^cells cultured in a DMEM medium supplemented with 15% FCS. The proliferation of SP and NSP cells in vitro were observed and assessed by proliferative curve (Figure 6A). The results showed that SP and NSP cells were adhesively cultured and presented with similar proliferative profiles in vitro. To further determine that the sorting SP fraction by FACS was an available method to enrich for Msi1 positive cells in vitro, SP and NSP cells sorted from Msi1^high ^cells were hypodermically engrafted into the backs of NOD/SCID mice. Two weeks after injection, grafts were developed with a diameter of 1 to 1.5 cm.

The grafts developed from SP and NSP both contained a mixture of well-differentiated tissues and many immature cells without specific structure. Abundant adenoid (AD), neural tube-like (NT), and sack-like structures were observed in the grafts from SP cells (Figure 6B). Fibrous tissues, macroscopic cartilages, nest-like structures, and pigment epithelium were observed in NSP grafts (Figure 6B).

Because Msi1 is regarded as a marker for IESCs and NSCs, the development of intestinal epithelial and neural cells in SP grafts were investigated [21,47,48]. The mRNA expressions of neural tissue markers (Nestin and Tubulin β III) and intestinal epithelial cells markers (SI and Fabp2 for absorptive cells; Tff3 for goblet cells; Lyz1 for Paneth cells; ChgA for endocrine cells) were detected in grafts from SP and NSP cells by RT-PCR (Figure 6C). The results revealed that the mRNA relative abundance was 0.681 ± 0.108 for Nestin, 0.703 ± 0.112 for Tubulin β III, 0.357 ± 0.061 for SI, 0.634 ± 0.091 for Fabp2, 0.077 ± 0.017 for Tff3, 0.218 ± 0.034 for Lyz1, and 0.068 ± 0.016 for ChgA in SP grafts (Figure 6D, E). The mRNA abundance in NSP grafts was 0.101 ± 0.027 for Lyz1 and 0.072 ± 0.017 for ChgA (Figure 6E). The expressions of Nestin, Tubulin β III, SI, Fabp2, and Tff3 were not detected in NSP grafts. The statistical analysis revealed that the mRNA expressions of Nestin, Tubulin β III, SI, Fabp2, Tff3, and Lyz1 in SP grafts were significantly higher than that in NSP grafts (*P*< 0.05). These data indicated that more neural tissues and small intestinal epithelial cells were developed in the grafts from SP cells.

To further characterize the developed potential of SP cells from Msi1^high ^cells, grafts were immunohistochemically stained with Tubulin β III and Fabp2 to detect the differentiation of neural and small intestinal epithelial tissues in vivo. The results showed that the Tubulin β III positive cells were located in some of nonspecific structures and nest-like structures (Figure 6F). More Tubulin β III-positive cells were detected in the grafts from SP cells than in grafts from NSP cells (Figure 6F). Fabp2 is a marker protein for intestinal absorptive cells. The results revealed that more Fabp2 positive cells were detected in the grafts from SP cells (Figure 6F). Partial Fabp2 positive cells formed sack-like structures. These special structures, mostly constructed of monolayer cells, were similar to the intestinal crypt structure of fetal mice (Figure 6F). These results indicated that SP cells sorted from Msi1^high ^cells had similar differentiated potentials with Msi1 positive cells, which could develop into neural and intestinal epithelial tissues in vivo [24].

## Discussion

The therapeutic potential of ESC-derived stem cells has been hindered by the formation of teratomas. Purifying ESC-derived stem cells is a potential approach to overcome this barrier. SP cells are identified and isolated in many different tissues, tumors, and cell lines, and are generally accepted as a unique character for stem cells [49]. The percentages of SP cells derived from ESCs range from 1% to 16% of total viable cells, depending on the stage of ESC development [50]. In the current study, SP cells were identified and sorted in the induction stage cells from mouse ESCs cultured in EGF, SF, and FCS medium, and the SP percentage in EGF group was significantly higher than that in control groups. Consisted with our previous findings showing that EGF can induce the differentiation of ESCs into Msi1^high ^cells and increase the percentage of Msi1 positive cells, 5-day EGF administration enhanced the percentage of SP cells (Figure 2C), suggesting that the SP fraction probably contains a large proportion of stem or progenitor cells derived from ESCs, including the Msi1 positive cells [23].

Until recently, it was impossible to isolate IESCs and NSCs based on identifying any single marker expressed on the cell surface. As a protein in the cytoplasm and nucleus required for asymmetric cell division, Msi1 is expressed in NSCs as well as in IESCs [51]. In our previous study, the Msi1-positive cells sorted from ESC-derived cells after a pMsi1-GFP vector transfection had the potential to differentiate into neural and intestinal epithelial cells in vivo [24]. However, the isolation process is complicated and completely depends on cell transfection, which restricts its application. It was reported by Dekaney et al that the SP fraction sorted from mouse jejunum had the stem-like characters and highly expressed Msi1 [36]. SP cells sorted from colon also expressed Msi1, β-integrin, and CD133. Consist with these previous studies, our results demonstrated that SP fraction contained almost all Msi1-positive cells (Figure 3B, D), indicating that sorting SP fraction by FACS, which is regarded as an effective and convenient method to enrich for stem or progenitor cells, could be a reliable method to enrich for Msi1-positive cells from the differentiated cell population derived from ESCs.

Zhou et al found that ABCG2, a subtype member of the ATP binding cassette (ABC) transporter, is a molecular determinant of the SP phenotype in mouse bone marrow [52,53]. Furthermore, ABCG2 expression was also identified in SP cells sorted from other tissues, such as skeletal muscle, liver, mammary gland, lung, and skin [27,30,32,33,41]. These studies demonstrated that ABCG2 plays an important role in the SP phenotype. However, it had become clear that the expression of ABCG2 was not detected in the all SP cells. The NSP cells sorted from mouse ESCs expressed Bcrp1 at a level equivalent to that from the SP fraction [53]. It was reported by Alt et al that ABCG2 expression was not detected in the SP cells from human umbilical cord blood [54]. This discrepancy was also reported in mammary gland cells and haemopoietic cells [55,56]. In our study, the SP phenotype is partially caused by ABCG2 activity, as evidenced by the marked reduction in SP cells with the administration of verapamil, an inhibitor of the ABCG2, which can block the formation of the SP fraction. The results showed that a similar expression of ABCG2 between SP and NSP fraction sorted from Msi1^high ^cells was detected, indicating that ABCG2 expression is not sufficient to confer the SP phenotype (Figure 5A, B). Because there is a significant overlap in the substrate specificity of ABC transporters, with each of the commonly studied family members ABCG2, MDR1, and MRP1 being capable of effluxing Hoechst 33342 dye, the mechanism contributing to SP phenotype sorted from Msil^high ^cells should be further investigated [55,56].

Msi1, a marker for NSC and IESC, plays key roles in the maintenance of the stem cell state and its differentiation, which had been shown by several scholars and us [20-22,57]. The Msi1 protein can also be found in tissues from patients with endometriosis and endometrial carcinoma, photoreceptor cells, retinal stem cells, and the hair follicle stem cell nich [58,59]. To confirm that the SP cells sorted from Msil^high ^cells had the phenotype of NSC and IESC, the expressions of other markers for IESCs (Lgr5, Hes1, Bmi1, and Ascl2) and NSCs (Sox2 and Nestin) in SP and NSP fractions were detected, respectively (Figure 4) [42-46]. The results revealed that sorting SP fraction could enrich for Lgr5, Hes1, Bmi1, and Nestin high expression cells, further indicating that SP cells could have the developed potentials of IESCs and NSCs. Recent studies showed that IESCs could be grouped into two different phenotypes [22]. One group presented with Msi1 and Hes1 expression resides in intestinal crypts near the transit-amplifying cells. Another marked with Lgr5 and Ascl2 resides between Paneth cells at the small intestinal crypt base. However, Ascl2 expression in SP fraction was similar with NSP fraction and not consistent with the expression of Lgr5 (Figure 4A, D). The reason for the inconsistent expression between Lgr5 and Ascl2 is not clear and should be further investigated.

As mentioned above, the strong expression of Msi1 and other markers for IESCs and NSCs were detected in SP fraction. SP cells sorted from Msi1^high ^cells were engrafted into the NOD/SCID mice to illuminate their developed profiles compared with NSP cells in vivo. The results showed that the SP grafts tended to differentiate into AD-like, NT-like, and sack-like structures (Figure 6A). This pathological profile suggested that SP graft contained more tissues and structures developed from NSCs and IESCs. Consistent with this observation, the expression of neural tissue markers (Nestin and Tubulin β III) and intestinal epithelial cells markers (Tubulin β III, SI, Fabp2, Tff3, and Lyz1) were significantly higher in SP grafts compared with NSP grafts (Figure 6B-6D). In addition, immunochemistry showed that more Tubulin β III positive cells partially constructed with a nest-like profile and more Fabp2-positive cells were observed in the SP grafts (Figure 6E). These results provided strong evidence that SP cells sorted from Msi1^high ^cells had the similar potential of Msi1 positive cells, which could develop into mature neural and intestinal epithelial tissues in vivo. Because forming grafts in NOD/SCID mice is a method for investigating the developed potential of the Msi1-positive cells in vivo, we have not yet performed the experiments necessary to prove that the cells isolated from Msi1^high ^cells will not form teratomas in the mice with normal immune function.

## Conclusions

In conclusion, SP was identified and isolated from Msi1^high ^cell population derived from ESCs in vitro in this study. Furthermore, the sorted SP fraction contains almost all the Msi1 positive cells and has the potential to differentiate into neural and intestinal epithelial cells in vivo. Therefore, sorting SP fraction could be a convenient and effective method to enrich for Msi1-positive cells from the differentiated cell population derived from mouse ESCs.

## Abbreviation

(**ESC**): embryonic stem cell; (**Msi1**): Musashi1; (**IESC**): intestinal epithelial stem cell; (**NSCs**): neural stem cells; (**Hes1**): hairy and enhancer of split 1; (**SP**): side population; (**FACS**): fluorescence activated cell sorter; (**EB**): embryonic body; (**DMEM**): Dulbecco's Modified Eagle Medium; (**FCS**): fetal calf serum; (**LIF**): leukemia inhibitory factor; (**KSR**): Knockout™ serum replacement; (**Lgr5**): leucine rich repeat containing G protein coupled receptor 5; (**Ascl2**): achaete-scute complex homolog 2; (**Sox2**): SRY-box containing gene 2; (**PI**): propidium iodide; (**NSP**): non-side population; (**ABCG2**): ATP-binding cassette transporter G2; (**SI**): sucrase-isomaltase; (**Fabp2**): fatty acid binding protein 2; (**Tff3**): trefoil factor 3; (**Lyz1**): lysozyme1; (**ChgA**): Chromogranin A; (**DAB**): 3,3'-diaminobenzidine tetrahydrochloride; (**SE**): standard error; (**AD**): adenoid; (**NT**): neural tube-like; (**ABC**): ATP binding cassette.

## Authors' contributions

TY and LNZ carried out the molecular genetic studies, participated in the sequence alignment and drafted the manuscript. SYL carried out the immunoassays. MJF participated in the pathological analysis. YG and LS participated in the sequence alignment. YHY and KHH participated in the design of the study and performed the statistical analysis. QKC conceived of the study, and participated in its design and coordination and helped to draft the manuscript. All authors read and approved the final manuscript.

## References

[B1] Yu J Thomson JA Pluripotent stem cell lines Genes Dev 2008 22 1987 1997 10.1101/gad.1689808 18676805 PMC2735345

[B2] Keller G Embryonic stem cell differentiation: emergence of a new era in biology and medicine Genes Dev 2005 19 1129 1155 10.1101/gad.1303605 15905405

[B3] Li G Luo R Zhang J Yeo KS Xie F Way Tan EK Caille D Que J Kon OL Salto-Tellez M Meda P Lim SK Derivation of functional insulin-producing cell lines from primary mouse embryo culture Stem Cell Res 2009 2 29 40 10.1016/j.scr.2008.07.004 19383407

[B4] Touboul T Hannan NR Corbineau S Martinez A Martinet C Branchereau S Mainot S Strick-Marchand H Pedersen R Di Santo J Weber A Vallier L Generation of functional hepatocytes from human embryonic stem cells under chemically defined conditions that recapitulate liver development Hepatology 2010 51 1754 1765 10.1002/hep.23506 20301097

[B5] Cao N Liao J Liu Z Zhu W Wang J Liu L Yu L Xu P Cui C Xiao L Yang HT In vitro differentiation of rat embryonic stem cells into functional cardiomyocytes Cell Res 2011 21 1316 1331 10.1038/cr.2011.48 21423272 PMC3193466

[B6] Zhang WJ Park C Arentson E Choi K Modulation of hematopoietic and endothelial cell differentiation from mouse embryonic stem cells by different culture conditions Blood 2005 105 111 114 10.1182/blood-2004-04-1306 15231577

[B7] Karki S Pruszak J Isacson O Sonntag KC ES cell-derived neuroepithelial cell cultures J Vis Exp 2006 30 118 10.3791/118 PMC2504447 18704173

[B8] Chinzei R Tanaka Y Shimizu-Saito K Hara Y Kakinuma S Watanabe M Teramoto K Arii S Takase K Sato C Terada N Teraoka H Embryoid-body cells derived from a mouse embryonic stem cell line show differentiation into functional hepatocytes Hepatology 2002 36 22 29 12085345 10.1053/jhep.2002.34136

[B9] Ko JY Lee HS Park CH Koh HC Lee YS Lee SH Conditions for tumor-free and dopamine neuron-enriched grafts after transplanting human ES cell-derived neural precursor cells Mol Ther 2009 17 1761 1770 10.1038/mt.2009.148 19603007 PMC2835006

[B10] Takahashi K Mitsui K Yamanaka S Role of ERas in promoting tumour-like properties in mouse embryonic stem cells Nature 2003 423 541 545 10.1038/nature01646 12774123

[B11] Fujikawa T Oh SH Pi L Hatch HM Shupe T Petersen BE Teratoma formation leads to failure of treatment for type I diabetes using embryonic stem cell-derived insulin-producing cells Am J Pathol 2005 166 1781 1791 10.1016/S0002-9440(10)62488-1 15920163 PMC1602425

[B12] Hentze H Graichen R Colman A Cell therapy and the safety of embryonic stem cell-derived grafts Trends Biotechnol 2007 25 24 32 10.1016/j.tibtech.2006.10.010 17084475

[B13] Lin Q Fu Q Zhang Y Wang H Liu Z Zhou J Duan C Wang Y Wu K Wang C Tumourigenesis in the infarcted rat heart is eliminated through differentiation and enrichment of the transplanted embryonic stem cells Eur J Heart Fail 2010 12 1179 1185 10.1093/eurjhf/hfq144 20817694

[B14] Caspi O Huber I Kehat I Habib M Arbel G Gepstein A Yankelson L Aronson D Beyar R Gepstein L Transplantation of human embryonic stem cell-derived cardiomyocytes improves myocardial performance in infarcted rat hearts J Am Coll Cardiol 2007 50 1884 1893 10.1016/j.jacc.2007.07.054 17980256

[B15] Heo J Factor VM Uren T Takahama Y Lee JS Major M Feinstone SM Thorgeirsson SS Hepatic precursors derived from murine embryonic stem cells contribute to regeneration of injured liver Hepatology 2006 44 1478 1486 10.1002/hep.21441 17133486

[B16] Chaudhry GR Fecek C Lai MM Wu WC Chang M Vasquez A Pasierb M Trese MT Fate of embryonic stem cell derivatives implanted into the vitreous of a slow retinal degenerative mouse model Stem Cells Dev 2009 18 247 258 10.1089/scd.2008.0057 18442304

[B17] Schriebl K Lim S Choo A Tscheliessnig A Jungbauer A Stem cell separation: A bottleneck in stem cell therapy Biotechnol J 2010 5 50 61 10.1002/biot.200900115 19946874

[B18] Konuma N Wakabayashi K Matsumoto T Kusumi Y Masuko T Iribe Y Mitsumata M Okano H Kusafuka T Mugishima H Mouse embryonic stem cells give rise to gut-like morphogenesis, including intestinal stem cells, in the embryoid body model Stem Cells Dev 2009 18 113 126 10.1089/scd.2008.0045 18680392

[B19] Torihashi S Kuwahara M Ogaeri T Zhu P Kurahashi M Fujimoto T Gut-like structures from mouse embryonic stem cells as an in vitro model for gut organogenesis preserving developmental potential after transplantation Stem Cells 2006 24 2618 2626 10.1634/stemcells.2006-0148 16888283

[B20] Kaneko Y Sakakibara S Imai T Suzuki A Nakamura Y Sawamoto K Ogawa Y Toyama Y Miyata T Okano H Musashi1: an evolutionally conserved marker for CNS progenitor cells including neural stem cells Dev Neurosci 2000 22 139 153 10.1159/000017435 10657706

[B21] Potten CS Booth C Tudor GL Booth D Brady G Hurley P Ashton G Clarke R Sakakibara S Okano H Identification of a putative intestinal stem cell and early lineage marker, musashi-1 Differentiation 2003 71 28 41 10.1046/j.1432-0436.2003.700603.x 12558601

[B22] Montgomery RK Breault DT Small intestinal stem cell markers J Anat 2008 213 52 58 10.1111/j.1469-7580.2008.00925.x 18638070 PMC2475558

[B23] Yu T Lan SY Wu B Pan QH Shi L Huang KH Lin Y Chen QK Musashi1 and hairy and enhancer of split 1 high expression cells derived from embryonic stem cells enhance the repair of small intestinal injury in the mouse Dig Dis Sci 2011 56 1354 1368 10.1007/s10620-010-1441-9 21221806

[B24] Lan SY Yu T Xia ZS Yuan YH Shi L Lin Y Huang KH Chen QK Musashi 1-positive cells derived from mouse embryonic stem cells can differentiate into neural and intestinal epithelial-like cells in vivo Cell Biol Int 2010 34 1171 1180 10.1042/CBI20100108 20670215

[B25] Wu C Alman BA Side population cells in human cancers Cancer Letters 2008 268 1 9 10.1016/j.canlet.2008.03.048 18487012

[B26] Goodell MA Brose K Paradis G Conner AS Mulligan RC Isolation and functional properties of murine hematopoietic stem cells that are replicating in vivo J Exp Med 1996 183 1797 1806 10.1084/jem.183.4.1797 8666936 PMC2192511

[B27] Yano S Ito Y Fujimoto M Hamazaki TS Tamaki K Okochi H Characterization and localization of side population cells in mouse skin Stem Cells 2005 23 834 841 10.1634/stemcells.2004-0226 15917479

[B28] Larderet G Fortunel NO Vaigot P Cegalerba M Maltère P Zobiri O Gidrol X Waksman G Martin MT Human side population keratinocytes exhibit long-term proliferative potential and a specific gene expression profile and can form a pluristratified epidermis Stem Cells 2006 24 965 974 10.1634/stemcells.2005-0196 16282445

[B29] Majka SM Beutz MA Hagen M Izzo AA Voelkel N Helm KM Identification of novel resident pulmonary stem cells: form and function of the lung side population Stem Cells 2005 23 1073 1081 10.1634/stemcells.2005-0039 15987674

[B30] Martin CM Meeson AP Robertson SM Hawke TJ Richardson JA Bates S Goetsch SC Gallardo TD Garry DJ Persistent expression of the ATP-binding cassette transporter, Abcg2, identifies cardiac SP cells in the developing and adult heart Dev Biol 2004 265 262 275 10.1016/j.ydbio.2003.09.028 14697368

[B31] Kim M Morshead CM Distinct populations of forebrain neural stem and progenitor cells can be isolated using side-population analysis J Neurosci 2003 23 10703 1079 14627655 10.1523/JNEUROSCI.23-33-10703.2003 PMC6740907

[B32] Shimano K Satake M Okaya A Kitanaka J Kitanaka N Takemura M Sakagami M Terada N Tsujimura T Hepatic oval cells have the side population phenotype defined by expression of ATPbinding cassette transporter ABCG2/BCRP1 Am J Pathol 2003 163 3 9 10.1016/S0002-9440(10)63624-3 12819005 PMC1868160

[B33] Alvi AJ Clayton H Joshi C Enver T Ashworth A Vivanco MM Dale TC Smalley MJ Functional and molecular characterisation of mammary side population cells Breast Cancer Res 2003 5 R1 R8 12559051 10.1186/bcr547 PMC154129

[B34] Behbod F Xian W Shaw CA Hilsenbeck SG Tsimelzon A Rosen JM Transcriptional profiling of mammary gland side population cells Stem Cells 2006 24 1065 1074 10.1634/stemcells.2005-0375 16282442

[B35] Meeson AP Hawke TJ Graham S Jiang N Elterman J Hutcheson K Dimaio JM Gallardo TD Garry DJ Cellular and molecular regulation of skeletal muscle side population cells Stem Cells 2004 22 1305 1320 10.1634/stemcells.2004-0077 15579648

[B36] Dekaney CM Rodriguez JM Graul MC Henning SJ Isolation and characterization of a putative intestinal stem cell fraction from mouse jejunum Gastroenterology 2005 129 1567 1580 10.1053/j.gastro.2005.08.011 16285956

[B37] Schmittgen TD Zakrajsek BA Mills AG Gorn V Singer MJ Reed MW Quantitative reverse transcription-polymerase chain reaction to study mRNA decay: comparison of endpoint and real-time methods Anal Biochem 2000 285 194 204 10.1006/abio.2000.4753 11017702

[B38] Goodell MA Brose K Paradis G Conner AS Mulligan RC Isolation and fuctional properties of murine hematopoietic stem cells that are replicating in vivo J Exp Med 1996 183 1797 1806 10.1084/jem.183.4.1797 8666936 PMC2192511

[B39] Park KS Lim CH Min BM Lee JL Chung HY Joo CK Park CW Son Y The side population cells in the rabbit limbus sensitively increased in response to the central cornea wounding Invest Ophthalmol Vis Sci 2006 47 892 900 10.1167/iovs.05-1006 16505021

[B40] Giangreco A Shen H Reynolds SD Stripp BR Molecular phenotype of airway side population cells Am J Physiol Lung Cell Mol Physiol 2004 286 L624 L630 12909587 10.1152/ajplung.00149.2003

[B41] Summer R Kotton DN Sun X Ma B Fitzsimmons K Fine A Side population cells and Bcrp1 expression in lung Am J Physiol Lung Cell Mol Physiol 2003 285 L97 L104 12626330 10.1152/ajplung.00009.2003

[B42] Snippert HJ van der Flier LG Sato T van Es JH van den Born M Kroon-Veenboer C Barker N Klein AM van Rheenen J Simons BD Clevers H Intestinal crypt homeostasis results from neutral competition between symmetrically dividing Lgr5 stem cells Cell 2010 143 134 144 10.1016/j.cell.2010.09.016 20887898

[B43] Reinisch C Kandutsch S Uthman A Pammer J BMI-1: a protein expressed in stem cells, specialized cells and tumors of the gastrointestinal tract Histol Histopathol 2006 21 1143 1149 16874656 10.14670/HH-21.1143

[B44] Jubb AM Chalasani S Frantz GD Smits R Grabsch HI Kavi V Maughan NJ Hillan KJ Quirke P Koeppen H Achaete-scute like 2 (ascl2) is a target of Wnt signalling and is upregulated in intestinal neoplasia Oncogene 2006 25 3445 3457 10.1038/sj.onc.1209382 16568095

[B45] Takemoto T Uchikawa M Yoshida M Bell DM Lovell-Badge R Papaioannou VE Kondoh H Tbx6-dependent Sox2 regulation determines neural or mesodermal fate in axial stem cells Nature 2011 470 394 398 10.1038/nature09729 21331042 PMC3042233

[B46] Park D Xiang AP Mao FF Zhang L Di CG Liu XM Shao Y Ma BF Lee JH Ha KS Walton N Lahn BT Nestin is required for the proper self-renewal of neural stem cells Stem Cells 2010 28 2162 2171 10.1002/stem.541 20963821

[B47] Asai R Okano H Yasugi S Correlation between Musashi-1 and c-hairy-1 expression and cell proliferation activity in the developing intestine and stomach of both chiken and mouse Develop Growth Differ 2005 47 501 510 10.1111/j.1440-169X.2005.00825.x 16287482

[B48] Kayahara T Sawada M Takaishi S Fukui H Seno H Fukuzawa H Suzuki K Hiai H Kageyama R Okano H Chiba T Candidate markers for stem and early progenitor cells, Musashi-1 and Hes-1, are expressed in crypt base columnar cells of mouse small intestine FEBS Lett 2003 535 131 135 10.1016/S0014-5793(02)03896-6 12560091

[B49] Challen GA Little MH A side order of stem cells: the SP phenotype Stem Cells 2006 24 3 12 10.1634/stemcells.2005-0116 16449630

[B50] Nadin BM Goodell MA Hirschi KK Phenotype and hematopoietic potential of side population cells throughout embryonic development Blood 2003 102 2436 2443 10.1182/blood-2003-01-0118 12805065

[B51] Samuel S Walsh R Webb J Robins A Potten C Mahida YR Characterization of putative stem cells in isolated human colonic crypt epithelial cells and their interactions with myofibroblasts Am J Physiol Cell Physiol 2009 296 C296 C305 19073897 10.1152/ajpcell.00383.2008 PMC2643851

[B52] Zhou S Schuetz JD Bunting KD Colapietro AM Sampath J Morris JJ Lagutina I Grosveld GC Osawa M Nakauchi H Sorrentino BP The ABC transporter Bcrp1/ABCG2 is expressed in a wide variety of stem cells and is a molecular determinant of the side-population phenotype Nat Med 2001 7 1028 1034 10.1038/nm0901-1028 11533706

[B53] Zhou S Morris JJ Barnes Y Lan L Schuetz JD Sorrentino BP Bcrp1 gene expression is required for normal numbers of side population stem cells in mice, and confers relative protection to mitoxantrone in hematopoietic cells in vivo Proc Natl Acad Sci USA 2002 99 12339 12344 10.1073/pnas.192276999 12218177 PMC129446

[B54] Alt R Wilhelm F Pelz-Ackermann O Egger D Niederwieser D Cross M ABCG2 expression is correlated neither to side population nor to hematopoietic progenitor function in human umbilical cord blood Exp Hematol 2009 37 294 301 10.1016/j.exphem.2008.09.015 19101070

[B55] Jonker JW Freeman J Bolscher E Musters S Alvi AJ Titley I Schinkel AH Dale TC Contribution of the ABC Transporters Bcrp1 and Mdr1a/1b to the Side Population Phenotype in Mammary Gland and Bone Marrow of Mice Stem Cells 2005 23 1059 1065 10.1634/stemcells.2005-0150 16002779

[B56] Naylor CS Jaworska E Branson K Embleton MJ Chopra R Side population/ABCG2 -positive cells represent a heterogeneous group of haemopoietic cells: implications for the use of adult stem cells in transplantation and plasticity protocols Bone Marrow Transplant 2005 35 353 360 10.1038/sj.bmt.1704762 15608658

[B57] Yu T Chen QK Gong Y Xia ZS Royal CR Huang KH Higher expression patterns of the intestinal stem cell markers Musashi-1 and hairy and enhancer of split 1 and their correspondence with proliferation patterns in the mouse jejunum Med Sci Monit 2010 16 BR68 BR74 20110912

[B58] Kaneko J Chiba C Immunohistochemical analysis of Musashi-1 expression during retinal regeneration of adult newt Neurosci Lett 2009 450 252 257 10.1016/j.neulet.2008.11.031 19028551

[B59] Götte M Wolf M Staebler A Buchweitz O Kelsch R Schüring AN Kiesel L Increased expression of the adult stem cell marker Musashi-1 in endometriosis and endometrial carcinoma J Pathol 2008 215 317 329 10.1002/path.2364 18473332

